# Tracking and Decomposing Health and Disease Inequality in Thailand

**DOI:** 10.1016/j.annepidem.2009.04.009

**Published:** 2009-11

**Authors:** Vasoontara Yiengprugsawan, Lynette L.-Y. Lim, Gordon A. Carmichael, Sam-Ang Seubsman, Adrian C. Sleigh

**Affiliations:** a Australian National University, ANU College of Medicine, Biology and Environment, National Centre for Epidemiology and Population Health, Canberra, Australia; b Thai Health-Risk Transition: a National Cohort Study, Sukhothai Thammathirat Open University, Nonthaburi, Thailand

**Keywords:** Concentration index, Decomposition, Health inequality, Specific diseases, Thailand, HWS, Health and Welfare Surveys, NSO, National Statistical Office

## Abstract

**Purpose:**

In middle-income countries, interest in the study of inequalities in health has focused on aggregate types of health outcomes, like rates of mortality. This work moves beyond such measures to focus on disease-specific health outcomes with the use of national health survey data.

**Methods:**

Cross-sectional data from the national Health and Welfare Survey 2003, covering 52,030 adult aged 15 or older, were analyzed. The health outcomes were the 20 most commonly reported diseases. The age-sex adjusted concentration index (C∗) of ill health was used as a measure of socioeconomic health inequality (values ranging from −1 to +1). A negative (or positive) concentration index shows that a disease was more concentrated among the less well off (or better off). Crude concentration indices (C) for four of the most common diseases were also decomposed to quantify determinants of inequalities.

**Results:**

Several diseases, such as malaria (C∗ = −0.462), goiter (C∗ = −0.352), kidney stone (C∗ = −0.261), and tuberculosis (C∗ = −0.233), were strongly concentrated among those with lower incomes, whereas allergic conditions (C∗ = 0.174) and migraine (C∗ = 0.085) were disproportionately reported by the better off. Inequalities were found to be associated with older age, low education, and residence in the rural Northeast and rural North of Thailand.

**Conclusions:**

Pro-equity health policy in Thailand and other middle-income countries with health surveys can now be informed by national data combining epidemiological, socioeconomic and health statistics in ways not previously possible.

## Introduction

Studies of inequity in health in the developed world have evolved beyond merely measuring inequality and are now also beginning to identify and quantify its causes. Recent health economic literature has provided tools for quantifying contributions to inequalities of individual determinants (1–3). Being able to differentiate and grade the determinants of inequalities such as age, sex, ethnicity, education, occupation or geographic location are essential to the design and targeting of pro-equity health interventions.

Like many developing countries, Thailand faces the challenge of rapid economic growth and widening inequality. Economic disparity between different geographic areas has translated into differences in health outcomes (4–6). The Thai Ministry of Public Health's *Thailand Health Profile* reveals concern over rural−urban and regional variations in health outcomes and the unequal geographic distribution of health resources (7). Thailand attempted to address its concern over health inequalities by introducing in 2001 a Universal Coverage Scheme to provide health insurance for all. Since then, monitoring the impacts of this scheme on health status, use of health services, and healthcare expenditure has been a major challenge in advancing health system equity.

Constrained by data availability, recent studies of inequalities in health in developing countries have focused mainly on available aggregate measures by trhe use of indicators such as child mortality and malnutrition (8–10). However, national health survey data that can complement other types of routine health surveillance data now are becoming available. Such information has not yet been fully used for monitoring inequity. So far, only a few studies have reported the relative contributions to inequality (through “decomposition”) of various identified determinants, and these use aggregated surveillance data; for example, inequalities in malnutrition prevalence in Vietnam (1) or inequalities in infant mortality in Iran (11). Here, we also report from a developing country and use national health data, but we analyze inequity and decompose its determinants at the individual level. Moreover, we report from a country that, like many others, is now moving rapidly through demographic and epidemiological transitions that make it necessary to have a system for regularly updating data on health inequity.

We have three objectives: (i) to quantify the socioeconomic maldistribution of specific reported diseases in Thailand; (ii) to “decompose” these inequalities, quantifying the contributions of specific determinants; and (iii) to demonstrate use of a periodic national health survey to monitor inequity in a middle-income country.

## Methods

### Data Source

Household interview surveys are now common sources of data in developing countries (12). This study used the 2003 wave of the periodic Thai Health and Welfare Surveys (HWS) to conduct a cross-sectional analysis. The 2003 wave was the first HWS conducted by the Thai National Statistical Office (NSO) after implementation of the Universal Coverage Scheme in 2001. In these national health surveys, every available member of a participating sample household aged 15 or older was interviewed about his or her current and recent morbidity (including injuries and disabilities), health-seeking behavior, and illness expenditure.

Data for children were also obtained from parents but were not used in this study. Where available, proxy respondents provided data for household members ages 15 or older still absent and unavailable for personal interview after three visits at different times of the day and days of the week. These data covered factual items other adult household members might be expected to know but excluded such items as self-assessed general health status that could not plausibly be answered on behalf of another person. Proxies were ages 15 or older and selected in a preferential order, with, for example, a spouse or parent selected ahead of less immediate kin.

### Study Population

The 2003 HWS covered 19,952 households with 68,433 members. After excluding children aged less than 15 years, data for 52,030 individuals were analyzed. Data for 28.5 percent of these individuals were provided by proxy respondents. As for 26,520 households were sampled for the 2003 HWS, 16,680 in non-municipal or rural areas and 9,840 in municipal or urban areas, the response rate at household level was 75.2%. Nonresponding households will have been a mixture (in unknown proportions) of vacant dwellings, dwellings at which no contact could be made after three visits, and refusals to participate; the response rate will therefore have been greater than the previous figure among households at which contact was made. The cutoff age of 15 was fixed by the NSO and possibly reflects the fact that that is the age by which Thais generally have completed their compulsory schooling and may be entering the workforce.

### Sampling Technique

A multistage sampling method has been adopted for the HWS. Each of the 76 provinces was divided up into sampling units consisting of urban areas (blocks) and rural areas (villages). A random sample of blocks and villages was then chosen within each province with the size of the sample in proportion to the total population of the province. Then within each sampled block, 15 random households were chosen and within each sampled villages 12 households were chosen. Weights provided with the HWS enabled results representative of the Thai population to be produced.

### Variables

Reported diseases were coded with the use of the Thai NSO system, which records specific disease subcategories under 15 major categories. Diseases were probed in the context of asking respondents (i) whether they had suffered any recent illness (in the past month), (ii) whether they suffered from any chronic medical condition that had lasted for more than three months, and (iii) whether they had been hospitalized (for a nonmaternity reason) during the past 12 months. Upon responding affirmatively to any of these three questions they were asked “What was the kind of sickness or symptoms did you have?” followed by “How did you know that you had this condition?” The present analysis covers the 20 most frequently reported disease categories and these correspond reasonably well to general or specific conditions under the International Classification of Diseases, Revision 10 code.

Attributes examined to potentially explain observed socioeconomic concentrations of the reported diseases were as follows: (i) age−sex interaction; (ii) household type; (iii) socioeconomic status (level of education and occupation/economic activity status); and (iv) geographic location, i.e., a rural−urban and region interaction variable.

### Statistical Analysis

All statistical analyses were performed with the use of STATA version 9 (13). Two-tailed tests reporting statistically significant results are highlighted (∗*p* < 0.05 †*p* < 0.01, and ‡*p* < 0.001). Correlations between determinants did not show significant multi-collinearity (none of the correlation coefficients between variables were greater than 0.65), and this conclusion was supported by analysis of variance inflation factors. In all models, no variance inflation factor value was >12, only 2 variables had values between 9 and 10, and the rest were <4. Accordingly, all variables were retained in the model.

Inequalities in individual diseases are examined by way of age-sex-adjusted concentration indices (C∗), which control for age-sex-confounding in crude concentration indices (C) (14). Decomposition analyses were conducted on crude concentration indices because in these analyses age−sex categories assume the status of potential determinants of inequality. Both C and C∗ take on values ranging between −1 and +1, with 0 indicating no inequality and negative (positive) values indicating concentration among the less well off (better off). The greater the absolute value of C or C∗, the greater the degree of concentration is in a negative or positive direction.

Monthly adult-equivalent income household is used as the measure of socioeconomic status. For Thailand, empirical studies suggest weighting each child aged under 15 as 0.5 of an adult, and allowing for economies of scale applying to any household with more than one member by raising adult equivalent household size to the power of 0.75 (14, 15).

The age−sex structure of samples is known to be a confounder in the study of socioeconomic health inequalities. Elderly, for example, tend to have lower incomes, and because they are older, also tend to be sicker. Crude and age-sex adjusted concentration indices are calculated following the pioneer method of Kakwani et al. (16, 17):

(1) $$2\sigma_{R}^{2}\frac{y_{i}}{y}=\alpha +\beta R_{i}+{ɛ}_{i}$$

(2) $$2\sigma_{R}^{2}\left[\frac{y_{i}}{\bar{y}}-\frac{ŷ}{\bar{ŷ}}\right]=\alpha +\overset{ˆ}{\beta }R_{i}+{ɛ}_{i}$$

Where *y_i_* is the health (or other) outcome of interest for the *ith* person; $\bar{y}$ is its mean; *R_i_* is the fractional rank of an individual in the socioeconomic distribution from the most disadvantaged to the least disadvantaged; $\sigma_{R}^{2}$ is its variance; *ŷ*_*i*_ is the age-sex adjusted health outcome for the *ith* person; and ${\bar{ŷ}}_{i}$ is the mean of the age-sex adjusted health outcome. As a result of the regressions, *β* is the crude concentration index and $\overset{ˆ}{\beta }$ is the age-sex adjusted concentration index.

Using the decomposition approach, the crude concentration index can be expressed as the sum of contributions of various determinants.

(3) $$C=\sum_{k}\left(\frac{\theta_{k}\bar{x_{k}}}{\bar{y}}\right)C_{k}+\frac{GC_{s}}{\bar{y}}$$

The decomposition equation has two components. The first term is the explained component, in which θ_k_ is the coefficient of each determinant category *k* on the health outcome, $\bar{x_{k}}$ and *C_k_* are the mean and concentration index of each determinant category, and $\bar{y}$ is the mean of the health outcome. In the second term, the unexplained component *GC_ɛ_* is the generalized concentration index for the error term. This component cannot be explained by systematic variation in determinants across the socioeconomic distribution.

### Other Epidemiological Approaches to Measure Inequality

To facilitate understanding of the concentration index results, two more conventional epidemiological measures were calculated for two conditions, one concentrated among the less well off and the other among the better-off. The first measure is the rate difference (RD), which is the absolute difference in rates of morbidity between the lowest and highest quintiles (Q1−Q5). The second is the rate ratio (RR), which is the relative difference in morbidity rates between the lowest and highest quintiles (Q1/Q5).

## Results

### Age-Sex-adjusted Concentration Indices for 20 Commonly Reported Diseases

Table 1 shows the occurrence and age-sex-adjusted concentration index for each disease. Occurrence levels are shown as both raw frequencies in the HWS sample and percentages weighted to be nationally representative. Of the 20 conditions, 15 yielded negative concentration indices, of which 7 were significantly different from zero, indicating inequalities unfavorable to the less well off. Among the reported diseases, malaria (C∗ = −0.462), goiter (C∗ = −0.352), kidney stone (C∗ = −0.261), tuberculosis (C∗ = −0.233), skin problems (C∗ = −0.227), peptic ulcer (C∗ = −0.109), diarrhea (C∗ = −0.080), and musculoskeletal pain (C∗ = −0.077) were significantly concentrated among the less well off. Five conditions with positive concentration indices, meaning that they were reported more frequently by greater income groups, were  allergic conditions (C∗ = 0.174) and hypertension (C∗ = 0.028), which were significantly more concentrated among the better off, and migraine (C∗ = 0.085), thyroid (C∗ = 0.052), hypertension (C∗ = 0.028), and diabetes (C∗ = 0.006), which did not attain statistical significance. It is interesting to note that after adjusting for age−sex confounding (not shown), two illnesses had changed their signs (i.e., from C = −0.068 to C∗ = 0.028 for hypertension; and from C = −0.084 to C∗ = 0.006 for diabetes).

### “Poor−rich” Distribution of Potential Determinants

Crude concentration indices for potential determinants of health inequalities summarize the “poor−rich” distributions of explanatory variables in the sample (full list shown in Table 2). They show that the elderly, aged ≥60 years, were commonly poor (C = −0.236 for men, C = −0.244 for women), whereas those of working age (30−44) were mildly concentrated among the better off (C = 0.058 for men and C = 0.057 for women). Male one-person households tended to be relatively well off economically (C = 0.136), whereas the opposite was true of female one-person households (C = −0.196). Households with elderly (almost one-third of the sample after weighting to be nationally representative) and households with no working-age member (1.6% of the sample) were generally poorer households (C = −0.111 and C = −0.546, respectively).

Approximately three-fifths of the sample had primary education or less and were relatively poor (C = −0.145), whereas the almost 30% who had secondary education were better off economically (C = 0.111) and the 10% with greater education were extremely well off (C = 0.545). A little more than one fourth of respondents after weighting worked in agriculture or fishing and tended to be at the lower end of the socioeconomic distribution (C = −0.348), whereas professionals, technicians, and service workers were at the greater end of the distribution (C = 0.351). Concentration indices for geographic areas showed clearly the contrast between wealthy and less well-off areas. Fourteen percent of respondents lived in Bangkok, and they were a clearly advantaged group (C = 0.549). Comprising more than one fourth of the weighted sample, residents of the Northeast region living in rural areas were easily the worst off economically (C = −0.339), whereas those living in the rural North were a second relatively poor group (C = −0.257).

### Decomposition Results for Four Self-Reported Diseases

Table 3 presents a decomposition analysis of crude concentration indices for four commonly reported diseases including asthma (C = −0.089), hypertension (C = −0.068), peptic ulcer (C = −0.131), and musculoskeletal pain (C = −0.176). All four diseases are distributed unevenly by socioeconomic status and all four inequalities, as measured by crude concentration indices, were unfavorable to the poor. Table 3 displays the contribution of age and sex to the crude concentration indices while also adjusting for other demographic, socioeconomic, and geographic determinants. Percentages shown in Table 3 indicate proportional contributions of pro-poor determinants to the corresponding total explained negative concentration indices. They are percentages of the sum of that column's negative crude concentration index values.

Biological influences of age and sex played major explanatory roles across the four diseases, being particularly important for hypertension (20.0% of the explained component due to male elderly and 40.7% due to female elderly), asthma (21.4% due to male elderly and 10.9% due to female elderly), and musculoskeletal pain (13.2% due to male elderly and 27.9% due to female elderly).

Those with no better than primary education contributed appreciably to explained inequalities unfavourable to the poor for all four diseases: asthma 27.3%, peptic ulcer 18.5%, hypertension 16.8%, and musculoskeletal pain 15.5%. Working in the agricultural or fishery sector contributed 6.1% to inequality in musculoskeletal pain, a finding that probably attributable to the physical nature of work in this occupation category, which doubtless also leads some to leave the workforce due to disability (2.8% contribution).

Geographic determinants yielded some stark results. Holding everything else constant with urban Central as the reference category, residence in the rural Northeast was a major contributor to inequality in occurrence of peptic ulcer (34.1%), with residence in the rural North (20.3%) also a major factor. For asthma, living in the rural Northeast and rural North contributed 17.3% and 10.2%, respectively, to the inequality observed. For musculoskeletal diseases, living in these two areas contributed 10.3% and 15.0%, respectively, to the inequality disadvantageous to the poor. By contrast, inequality in hypertension owed very little to geographic location, and what it did owe was mainly due to residence in Bangkok.

Table 4 presents standard morbidity rates for musculoskeletal pain by income quintiles and age−sex groups. Rate differences and rate ratios between the highest and lowest quintiles are also calculated. A clear positive association between increasing age and musculoskeletal pain is evident for both sexes. There is also evidence of socioeconomic inequality in musculoskeletal pain, with the lowest income quintile associated with the highest disease rates and the highest quintile with the lowest morbidity rates across almost all age−sex groups. A similar analysis is presented for allergic conditions in Table 5. There is a notable difference in the direction of the socioeconomic inequality, with allergies tending to be disproportionately concentrated among the better-off.

## Discussion

Knowing the magnitude of socioeconomic inequalities is necessary but insufficient information on which to base health policy interventions; understanding the sources of inequalities has also become of great importance. Wagstaff et al. (1) and Hosseinpoor et al. (11) have shown that the decomposition method can be applied to quantify determinant-specific inequity in developing countries for aggregate outcomes such as child mortality and malnutrition prevalence. This paper applies similar methodology to a different set of informative health outcomes—reported occurrences of specific diseases—derived from interview data from a developing country's national health survey. The results confirm that in Thailand adverse health outcomes were mostly concentrated among the poor and associated with particular population subgroups. Furthermore, the contributions to inequalities of specific determinants have been quantified. Inequalities in specific reported diseases are associated with older age (often particularly in conjunction with being female), low education, and residence in the rural Northeast and rural North of Thailand.

Although the diseases and disorders covered in this study are self-reported, almost half of ill respondents were aware of their illnesses from medical diagnoses made by formal health services. However, some inequalities observed might be explained by health information and how it is used in different income groups. In some cases, reported illnesses may signify better access to health services at the better-off end of the socioeconomic spectrum (18, 19). For example, reporting hypertension or migraine may partly be attributed to the higher socioeconomic group visiting doctors more frequently, and therefore being able to attach these labels to their medical conditions. Thus, results can be biased by an inclination to underreport health problems that may be unknown to certain subgroups of respondents (20).

Given the nature of the secondary data analysis, one of the possible limitations of the study was its inability to adjust for the unmeasured confounding. Caution is also required in making causal links between reported diseases and potential determinants from the analysis. The design of the study is cross-sectional and, thus, data on outcomes and determinants were collected simultaneously. In addition, decomposition is a deterministic approach and only includes measured explanatory variables. There are clearly other determinants beyond the demographic, socioeconomic, and geographic characteristics covered, both within and outside the health system, which contribute to inequalities in health outcomes. For example, the disproportionate frequency of peptic ulcer among rural North and Northeastern Thais could reflect the culinary culture and food safety situation in this region, or even variation in genetic susceptibility (21).

Future studies could usefully make use of cohort or other longitudinal data to monitor changes in socioeconomic inequalities in reported diseases as Thailand moves through the epidemiological transition (22, 23). This work demonstrates how existing national data sources can be used to describe and potentially track socioeconomic inequalities in health and how new insights can be gained by applying concentration index and decomposition methods in a developing country. Periodic national health surveys will be particularly useful in monitoring changes in the use of health services among disadvantaged socioeconomic and geographic subgroups as Thailand moves further into the Universal Health Coverage era (24–28). Future population health policy in Thailand and other middle-income countries with similar resources can be informed by ways not possible before by national data combining epidemiology, socioeconomics and health information.

## Figures and Tables

**Table 1:** Occurrence and concentration indices of the 20 most commonly reported diseases^a^

	Occurrence		Age-sex adjusted concentration index^b^	
Reported diseases	Unweighted frequency (*n* = 52, 030)	Weighted frequency (%)	Value	95% confidence interval
Allergic conditions	889	1.6	**0.174‡**	(0.107, 0.241)
Migraine	191	0.3	0.085	(–0.042, 0.213)
Thyroid	255	0.5	0.052	(–0.037, 0.142)
Hypertension	2741	3.6	0.028	(–0.006, 0.062)
Diabetes mellitus	1447	2.2	0.006	(–0.039, 0.051)
Gout	209	0.4	−0.012	(–0.169, 0.145)
Common cold	2532	4.8	−0.019	(–0.057, 0.018)
Anemia	94	0.2	−0.065	(–0.225, 0.094)
Hemorrhagic fever (dengue fever)	87	0.2	−0.070	(–0.222, 0.082)
Musculoskeletal pain	1580	2.7	**−0.077‡**	(−0.121, −0.033)
Diarrhea, food poisoning	334	0.6	−0.080	(–0.174, 0.014)
Asthma	622	1.1	−0.048	(–0.130, 0.035)
Renal failure	167	0.3	−0.096	(–0.235, 0.043)
Peptic ulcer	1277	2.3	**−0.109‡**	(−0.158, −0.060)
Cataract	153	0.2	−0.120	(−0.250, 0.011)
Skin conditions	143	0.3	**−0.227‡**	(−0.367, −0.087)
Tuberculosis	46	0.1	**−0.233∗**	(−0.465, −0.002)
Kidney stone	98	0.2	**−0.261‡**	(−0.406, −0.116)
Goiter	90	0.2	**−0.352‡**	(−0.515, −0.189)
Malaria	24	0.1	**−0.462†**	(−0.807, −0.117)

a Diseases reported as an outpatient in the past month, and/or as a current chronic condition that has lasted >3 months and/or as a condition requiring hospitalization in the past 12 months.

b If the concentration index equals 0, there is no poor-rich inequality in the distribution of that disease. However, a negative (positive) concentration index shows that the disease is more concentrated among the poor (rich). The further away a concentration index is from zero, the greater the extent of the inequality in that direction. Concentration indices differing from a value of 0 by statistically significant amounts are highlighted (∗*p* < 0.05 †*p* < 0.01, and ‡*p* < 0.001).

**Table 2:** Poor−rich distribution of determinants

	Occurrence		Concentration index	
	Unweighted frequency (*n* = 52 030)	Weighted (%)	Value	95% confidence interval
Demographic characteristics				
Age-sex				
Males aged 15-29	7,137	17.9	0.019	(0.001, 0.037)
Males aged 30-44	9,170	15.7	0.056	(0.040, 0.072)
Males aged 45-59	6,778	10.1	0.058	(0.040, 0.077)
Males aged 60+	4,977	5.8	−0.236	(−0.261, −0.210)
Females aged 15-29	6,701	17.4	0.027	(0.009, 0.045)
Females aged 30-44	7,685	15.6	0.057	(0.042, 0.072)
Females aged 45-59	5,800	10.5	−0.010	(−0.028, 0.007)
Females aged 60+	3,782	6.9	−0.244	(−0.268, −0.220)
Household				
One-person male household	983	1.7	0.136	(0.066, 0.206)
One-person female household	1,209	1.5	−0.196	(−0.260, −0.132)
Household with children but no elderly	31,973	65.3	0.065	(0.056, 0.075)
Household with elderly	16,697	30.0	−0.111	(−0.132, −0.091)
Household with only dependents	1,168	1.6	−0.546	(−0.633, −0.459)
Socioeconomic characteristics				
Education				
Primary level or less	31,796	60.9	−0.145	(−0.152, −0.138)
Secondary level	13,803	28.7	0.111	(0.097, 0.125)
Higher level	6,431	10.4	0.545	(0.516, 0.573)
Occupation				
Agriculture and fishery	11,362	27.8	−0.348	(−0.363, −0.334)
Elementary occupation^a^	4,513	9.1	0.049	(0.025, 0.074)
Others such as professionals	21,668	36.7	0.351	(0.343, 0.360)
Not in workforce: housewife	4,080	6.9	−0.067	(−0.095, −0.040)
Not in workforce: disabled	1,009	1.9	−0.329	(−0.387, −0.272)
Not in workforce: others, such as students	9,398	17.6	−0.144	(−0.165, −0.124)
Geographic characteristics				
Bangkok	2,985	13.7	0.549	(0.511, 0.588)
Urban Central	9,423	8.1	0.356	(0.320, 0.392)
Rural Central	7,403	15.2	0.105	(0.079, 0.132)
Urban North	6,400	3.8	0.070	(0.037, 0.102)
Rural North	4,667	14.5	−0.257	(−0.286, −0.227)
Urban Northeast	7,035	5.4	0.067	(0.035, 0.099)
Rural Northeast	5,332	26.9	−0.339	(−0.360, −0.318)
Urban South	4,739	2.8	0.224	(0.182, 0.265)
Rural South	4,046	9.5	−0.047	(−0.077, −0.018)

a Elementary occupations include the likes of street vendors, domestics, and nonagricultural laborers.

**Table 3:** Decomposition results: contributions of determinants to concentration indices (absolute value and percentage of total explanatory variables)^a^

	Asthma (*n* = 622)		Hypertension (*n* = 2741)		Peptic ulcer (*n* = 1277)		Musculoskeletal pain (*n* = 1580)	
Crude concentration index^b^	−0.089		−0.068		−0.131		−0.176	
Demographic characteristics								
Age-sex	CCI	%^c^	CCI	%	CCI	%	CCI	%
Males aged 30-44	−0.001	0.7	0.002		0.001		0.005	
Males aged 45-59	0.000		0.006		0.003		0.011	
Males aged 60+	−0.028	21.4	−0.037	20.0	−0.013	7.8	−0.052	13.2
Females aged 15-29	−0.001	0.6	0.000		0.002		−0.001	0.3
Females aged 30-44	0.002		0.004		0.007		0.007	
Females aged 45-59	0.000	0.2	−0.002	1.2	−0.001	0.4	−0.003	0.7
Females aged 60+	−0.014	10.9	−0.076	40.7	−0.025	15.6	−0.109	27.9
Household								
One-person male household	0.000	0.4	0.000		0.002		0.001	
One-person female household	−0.001	0.5	0.000	0.3	−0.004	2.5	−0.004	1.0
Household with elderly	−0.002	1.4	0.002		0.009		0.009	
Household with only dependents	0.009		−0.008	4.4	0.004		−0.015	3.9
Socioeconomic characteristics								
Education								
Education: primary level or less	−0.036	27.3	−0.031	16.8	−0.030	18.5	−0.061	15.5
Education: secondary level	0.007		0.002		0.001		0.004	
Occupation								
Work: agriculture and fishery	0.030		0.019		0.002		−0.024	6.1
Work: elementary occupation	0.000	0.0	−0.001	0.6	0.000		0.000	0.1
Not in workforce: housewife	0.000	0.2	−0.001	0.5	0.001		0.000	0.1
Not in workforce: disabled	−0.003	2.6	−0.004	2.2	0.002		−0.011	2.8
Not in workforce: others, such as students	0.001		−0.006	3.4	0.006		−0.009	2.4
Geographic characteristics								
Bangkok	−0.007	5.0	−0.011	5.7	0.001		0.020	
Rural Central	0.004		0.002		0.003		0.000	
Urban North	0.000		0.001		0.002		0.002	
Rural North	−0.013	10.2	−0.007	3.9	−0.033	20.3	−0.059	15.0
Urban Northeast	0.001		−0.001	0.3	0.002		0.000	
Rural Northeast	−0.023	17.3	0.050		−0.055	34.1	−0.040	10.3
Urban South	0.005		0.002		0.001		0.001	
Rural South	−0.002	1.3	0.001		−0.001	0.7	−0.002	0.6
Total explained	−0.073	100	−0.096	100	−0.114	100	−0.329	100
Residuals (unexplained)	−0.017		0.028		−0.018		0.154	

CCI = Contribution to Concentration Index.

a Reference groups were males aged 15–29; household with no elderly; work status: others including professionals, technicians, or service workers; and urban Central excluding Bangkok. Elementary occupations include the likes of street vendors, domestics, and nonagricultural laborers.

b Crude concentration indices are not adjusted for age and sex.

c Percentages shown are proportional contributions to the total explained negative concentration index.

**Table 4:** Rates per thousand respondents reporting musculoskeletal pain by income quintile and age-sex group, 2003

Income quintile	Male 15–44	Female 15–44	Male 45–59	Female 45–59	Male 60+	Female 60+	Overall	Age-sex adjusted
Bottom 20% (Q1)	12.8	14.8	62.7	62.5	82.4	110.0	71.1	71.1
(Q2)	11.9	11.3	32.4	66.8	75.9	98.2	65.7	65.7
(Q3)	8.5	7.3	27.0	53.8	56.1	104.3	58.6	58.6
(Q4)	8.2	8.3	21.4	40.3	52.3	96.3	54.3	54.3
Top 20% (Q5)	6.8	6.9	18.8	37.4	41.8	94.4	49.8	49.8
RD (Q1 – Q5)	6.0	7.9	44.0	25.1	40.7	15.6	41.4	21.3
RR (Q1 / Q5)	1.9	2.2	3.3	1.7	2.0	1.2	2.1	1.4
N	14,386	16,307	5,800	6,778	3,782	4,977	52,030	52,030

**Table 5:** Rates per thousand respondents reporting allergic conditions by income quintile and age-sex group, 2003

Income quintile	Male 15–44	Female 15–44	Male 45–59	Female 45–59	Male 60+	Female 60+	Overall	Age-sex adjusted
Bottom 20% (Q1)	10.9	11.2	7.8	20.6	7.4	6.4	10.7	12.2
(Q2)	10.8	17.3	7.2	14.4	3.8	8.7	12.1	12.3
(Q3)	10.5	23.9	11.8	22.8	14.0	6.2	16.5	16.2
(Q4)	17.3	29.1	15.2	18.2	20.6	6.1	20.8	20.2
Top 20% (Q5)	22.8	31.1	23.4	21.0	15.4	8.0	24.3	23.6
RD (Q1 – Q5)	−11.9	−19.8	−15.6	−0.4	−8.0	−1.6	−13.6	−11.4
RR (Q1 / Q5)	0.5	0.4	0.3	1.0	0.5	0.8	0.4	0.5
N	14,386	16,307	5,800	6,778	3,782	4,977	52,030	52,030
